# The Toys (Poetry)

**Published:** 1881-12

**Authors:** 


### THE TOYS.

My little son, who look'd from thoughtful eyes,
And moved and spoke in quiet grown-up wise,
Having my law the seventh time disobey'd,
I struck him, and dismiss'd
With hard words and unkiss'd,
His mother, who was patient, being dead.
Then, fearing lest his grief should hinder sleep,
I visited his bed,
But found him slumbering deep,
With darken'd eyelids, and their lashes yet
From his late sobbing wet.
And I, with moan,
Kissing away his tears, left others of my own:
For, on a table drawn beside his head,
He put, within his reach,
A box of counters, and a red-vein'd stone,
A piece of glass abraded by the beach,
And six or seven shells,
A bottle with blue bells,
And two French copper coins, ranged there with careful art,
To comfort his sad heart.
So when that night I pray'd
To God, I wept and said:
Ah, when at last we lie with tranced breath,
Not vexing Thee in death,
And Thou rememberest of what toys
We made our joys,
How weakly understood
Thy great commanded good,
Then, fatherly, not less
Than I whom thou has molded from the clay,
Thou'lt leave Thy wrath, and say,
"I will be sorry for their childishness."

THEODORE remarked, when Angelina's father shoved him off the doorstep, that the old gentleman had considerable push about him.

PLEASURE comes through industry, and not by self-indulgence and indolence. When one gets to love work, his life is a happy one.—*Ruskin.*

